# Supplementary material for: Incentive Cocaine‐Seeking Habits and Their Compulsive Manifestation Emerge After a Downregulation of the Dopamine Transporter in Astrocytes Across Functional Domains of the Striatum
Source: Eur J Neurosci. 2025 Mar 13;61(5):e70054. doi: 10.1111/ejn.70054 (PMC11906910; doi:10.1111/ejn.70054)
Supplement: Supplementary file 1 — Figure S1. Raw uncropped western blot images. Figure S2. Behavioural and molecular characterisation of instrumentally trained vs naive control animals. Table S1. Correlations table. [file EJN-61-0-s001.docx]

Supplementary information for:

Incentive cocaine-seeking habits and their compulsive manifestation emerge after a downregulation of the dopamine transporter in astrocytes across functional domains of the striatum.

Maxime Fouyssac^1^, Tristan Hynes^1^, Aude Belin-Rauscent^1^, Dhaval D. Joshi^1^, David Belin^1^

^1^Department of Psychology, University of Cambridge, Cambridge, CB2 3EB, UK

Correspondence should be addressed to:

Dr Maxime Fouyssac (mf539@cam.ac.uk) or Professor David Belin ([bdb26@cam.ac.uk](mailto:bdb26@cam.ac.uk))

**This PDF file includes:**

Figures S1

Figures S2

Table S1

**Figure S1. Raw uncropped western blot images.**

**A** - **B)** complete unedited western blot images related to data presented in **Figure 2** from micro-punched samples pertaining to the three experimental conditions CTL, FR1C and SOR. **C** - **D)** complete unedited western blot images related to data presented in **Figure 3** from cultured astrocytes samples pertaining to the three experimental conditions CTL, FR1C and SOR. DAT proteins are revealed at ~70/75 kDa (top panels) and actin at ~40/45 kDa (bottoms panels).

**Figure S2. Behavioural and molecular characterisation of instrumentally trained vs naive control animals.**

**A)** Rats ascribed to the CTL group and used to assess DAT protein levels from total tissue (Punch group, light grey) did not differ in their food self-administration performance from those ascribed to the CTL group used to assess DAT protein levels from astrocyte cultures (Astro group, purple). Due to the lack of variance in the latter, non-parametric Mann Whitney tests were used to compare the two groups over the course of the 10 daily sessions. No significant difference between groups was found at any time point (U<15, p>0.12 for all comparisons). The seemingly lower performance displayed by the Punch group over the first sessions was due to a single rat that experienced a slower acquisition. **B)** As anticipated, DAT protein levels in total tissue from the five striatal territories did not differ between rats trained to self-administer food (FR1F group) and experimentally naive rats (Naive group), allowing us to pool the data from both groups into the CTL group presented in **Figure 2**. **C)** Similarly, DAT protein levels in cultured astrocytes from the five striatal territories did not differ between rats trained to self-administer food (FR1F group) and experimentally naive rats (Naive group), allowing us to pool the data from both groups into the CTL group presented in **Figure 3**.

**Table S1. Correlations table.**

Correlations between behavioural measures (instrumental responding in self-administration, i.e., total active lever presses or exposure to cocaine, i.e., total cocaine infusions received) and DAT protein or mRNA levels from data presented in **Figure 2-4**. AL presses: Active lever presses
